# Supplementary material for: Synergistic effect of collagen and CXCL12 in the low doses on human platelet activation
Source: PLoS One. 2020 Oct 29;15(10):e0241139. doi: 10.1371/journal.pone.0241139 (PMC7595269; doi:10.1371/journal.pone.0241139)
Supplement: Attachment — Submitted filename: Plos One.docx [file pone.0241139.s001.docx]

The manuscript is general sheds light on an important aspect of platelet biology, one in which CXCL12 plays a critical role in synergizing with collagen. We do have some issues that need to be addressed.

1. The “English” in the manuscript requires a lot of work, in terms of grammar and typos.
2. Abstract implies CXCL12 acts via CXCR only, whereas introduction via CXCR4 and CXCR7. This appears contradictory.
3. Elaborate on why the sCD40L assay the co-stimulation lasted for 15 minutes, unlike many of the other experiments.
4. SB203580 did not completely block the co-stimulation effects, which suggests a role for another pathway. What is it? Can you answer that experimentally?
5. All of the Western blot data should be quantified.
6. Another relevant article not cited is by Karim et al, 2016 in BBA (PMID: 26628381). It should be cited.
