# Supplementary material for: Synergistic effect of collagen and CXCL12 in the low doses on human platelet activation
Source: PLoS One. 2020 Oct 29;15(10):e0241139. doi: 10.1371/journal.pone.0241139 (PMC7595269; doi:10.1371/journal.pone.0241139)
Supplement: Attachment — Submitted filename: Response to Reviewers.docx [file pone.0241139.s002.docx]

Our responses to the reviewer’s comments and the changes in the re-revised manuscript

(PONE-D-19-34102R1)

Authors: Daiki Nakashima, Takashi Onuma, Kumiko Tanabe, Yuko Kito, Kodai Uematsu, Daisuke Mizutani, Yukiko Enomoto, Masanori Tsujimoto, Tomoaki Doi, Rie Matsushima-Nishiwaki, Haruhiko Tokuda, Shinji Ogura, Toru Iwama, Osamu Kozawa and Hiroki Iida

Title: Synergistic effect of collagen and CXCL12 in the low doses on human platelet activation

Our responses to reviewers:

**Comments from the academic editor and reviewers:**

**- Academic Editor**

**Thank you for submitting your manuscript to PLOS ONE. After careful consideration, we feel that it has merit but does not fully meet PLOS ONE’s publication criteria as it currently stands. Therefore, we invite you to submit a revised version of the manuscript that addresses the points raised during the review process.**

**The manuscript reports the effect of collagen and the chemokine CXCL12 on platelet aggregation and release of growth factor PDGF-AB, cytokine sCD40L and phosphorylated HSP27. Although the hypothesis is interesting, this study remains preliminary and descriptive. Further experiments on the type of platelet activation and its responsive receptors (ADP, thrombin, GPVI) should be performed, and dose-response curves of the synergistic effects of the platelet agonist collagen and CXCL12 should be evaluated. In addition, the specific points from the reviewers need a point to point response.**

Our responses:

At first, we deeply appreciate your adequate and favorable evaluation for our manuscript. We totally agreed to your requests that further experiments on the type of platelet activation and its responsive receptors (ADP, thrombin, GPVI) should be performed, and dose-responsive curves of the synergistic effects of the platelet agonist collagen and CXCL12 should be evaluated. We also realized that the specific points from the reviewers need a point to point response. To address your comments, we performed additional experiments as follows:

PRP was stimulated by CXCL12 with collagen, thrombin receptor-activating peptide (TRAP; Bachem, Budendorf, Switzerland), ADP (Merck-Sigma-Aldrich, Darmstadt, Germany) or convulxin (an activator of GPVI) (Cayman Chemical, Ann Arbor, MI). The dose of each stimulator achieving a % transmittance of 10%-30% recorded by the aggregometer was adjusted individually.

We examined the effects of TRAP or ADP in their subthreshold doses with CXCL12 (10 ng/ml) on human platelet aggregation. We here used TRAP instead of thrombin since TRAP is a potent activator of thrombin receptors, protease-activated receptors (PARs) (Aoki T, et al. Thromb Haemost. 1998;79:1184-1190). As results using laser scattering system, when the platelets were stimulated in combination with TRAP (7 μM) and CXCL12 compared with TRAP alone, the increase of transmittance or the size ratio of large aggregates was not detected whereas the size ratios of small and medium aggregates were increased. The representative results are presented as Fig. 2A in the re-revised manuscript. In addition, when the platelets were stimulated in combination with ADP (0.5 μM) and CXCL12 compared with ADP alone, the increase of transmittance or the size ratio of large aggregates was not detected whereas the size ratios of small and medium aggregates were increased. The representative results are presented as Fig 2B in the re-revised manuscript. These findings suggest that the synergistic effects of thrombin or ADP with CXCL12 at subthreshold doses on platelet aggregation were hardly observed. Therefore, it is probable that the synergistic effect of CXCL12 on human platelet aggregation is specific to collagen. However, it has been reported that CXCL12 and TRAP as well as collagen synergistically stimulate platelet aggregation in mice (Karim ZA, et al. Biochim Biophys Acta 2016;1863:314-321). The discrepancy between the present and previous findings might be due to differences in species or experimental conditions.

To further define the receptor for collagen in the synergistic effect of collagen and CXCL12 on human platelet aggregation, we examined the platelet aggregation stimulated with a combination of CXCL12 and convulxin. Convulxin is a snake venom activator of GPVI but not integrin α2/β3 (Niedergang F, et al. Biochem Biophys Res Commun. 2000;273:246-250). We found that convulxin at a dose of 20 ng/ml had little effect on human platelet aggregation whereas 30 ng/ml of convulxin alone stimulated the aggregation. As results, the combination of convulxin (20 ng/ml) and CXCL12 (10 ng/ml) synergistically stimulated the platelet aggregation. Therefore, these findings suggest that GPVI among collagen receptors is involved in the synergistic effect with CXCL12 on human platelet aggregation. The representative results are presented as Fig. 3 in the re-revised manuscript.

We next evaluated the dose-response curves of the synergistic effects of collagen and CXCL12 in human platelet aggregation. We examined the effect of the simultaneous stimulation of various doses (0.05 μg/ml, 0.1 μg/ml and 0.2 μg/ml) of collagen with a fixed dose (10 ng/ml) of CXCL12 on the platelet aggregation. As results, whereas collagen at a dose of up to 0.1 μg/ml with CXCL12 hardly increased the transmittance or size ratio of large aggregates, collagen at a dose of 0.2 μg/ml with CXCL12 significantly induced platelet aggregation. The representative results are presented as Fig. 1A in the re-revised manuscript. We further examined the effect of the simultaneous stimulation of various doses (1 ng/ml, 3 ng/ml and 10 ng/ml) of CXCL12 with a fixed dose (0.2 μg/ml) of collagen on the platelet aggregation. As results, whereas CXCL12 up to 3 ng/ml with collagen had little effect on the transmittance or size ratio of large aggregates, CXCL12 at a dose of 10 mg/ml with collagen significantly induced platelet aggregation. The representative results are presented as Fig. 1B in the re-revised manuscript. From the dose-response reactions of collagen and CXCL12, it is probable that the synergistic effect of collagen and CXCL12 in the combination of 0.2 μg/ml of collagen and 10 ng/ml of CXCL12 is prominent to induce the platelet aggregation, whereas the combination of these stimulators at lower doses is insufficient.

We presented the data of additional experiments as Fig. 1, Fig. 2, Fig. 3 and Fig. 4, cited the above articles as Ref. 32, Ref. 37 and Ref. 33, respectively, and added the description in “Abstract”, “Materials and methods”, “Results” and “Discussion” in the re-re-revised manuscript. (page 2, line 38 to line 44, page 6, line 151 to page 7, line 158, page 8, line 188 to line 192, page 10, line 240 to line 252, page 11, line 266 to line 278, page 12, line 290 to line 298, page 23, line 567 to line 577)

In addition, we responded all of the specific points from the reviewers as described below.

**Reviewer 2**

**The manuscript authored by Nakashima and coworkers describes in vitro findings on the effect of collagen and the chemokine CXCL12 on platelet aggregation and release of growth factor PDGF-AB, cytokine sCD40L and phosphorylated HSP27. The authors found that primarily the combination of collagen and CXCL12 stimulate the release of the determined compounds via activation of p38-MAPK. The authors claim that the described mechanisms may contribute to vascular inflammation and atherogenesis. The hypothesis that the chemokine CXCL12 may be a prominent inflammatory mediator that synergistically induce platelet release reaction upon collagen-induced platelet activation is clear. The synergistically effect of platelet activation via inflammatory mediators is a timely issue that deserves intensive research work.　The major limitation of the present study is that at present it remains descriptive with limited exploration of the underlying mechanisms. The authors convincingly show that collagen plus CXCL12 induce activation of the MAPK p38 and release of PDGF-AB, cytokine sCD40L, and phosphorylated HSP27. However, it is not clear whether this is specific for collagen-stimulated platelets or also is found for platelets stimulated with other agonists such as ADP or thrombin. Further, the receptor for collagen in this context should be further defined. Is this a specific GPVI-mediated effect? Specific experiments with collagen-related peptide (CRP) or convulxin will help. Further, CXCL12 binds to both CXCR4 and CXCR7 receptors on platelets. The authors need to show which is the prominent receptor for this putative synergistic effect on platelet release reaction and aggregation. And antibody-based approach using neutralizing anti-CXCR4 or anti-CXCR7 antibodies might disclose this aspect.**

Our responses:

At first, we deeply appreciate your adequate and favorable evaluation for our manuscript. We totally agreed to your criticism that at present it remains descriptive with limited exploration of the underlying mechanisms. We also appreciate your suggestions about the additional experiments to be performed. To address your comments, we performed additional experiments as follows:

PRP was stimulated by CXCL12 with collagen, thrombin receptor-activating peptide (TRAP; Bachem, Budendorf, Switzerland), ADP (Merck-Sigma-Aldrich, Darmstadt, Germany) or convulxin (an activator of GPVI) (Cayman Chemical, Ann Arbor, MI). The dose of each stimulator achieving a % transmittance of 10%-30% recorded by the aggregometer was adjusted individually. When indicated, PRP was pretreated with anti-CXCR4 monoclonal antibody, anti-CXCR7 monoclonal antibody (R&D systems, Mineapolis, MN) or control IgG (Santa Cruz Biotechnology, Inc., Santa Cruz, CA) for 3 min, and then stimulated by CXCL12 and/or collagen.

We examined the effects of TRAP or ADP in their subthreshold doses with CXCL12 (10 ng/ml) on human platelet aggregation. We here used TRAP instead of thrombin since TRAP is a potent activator of thrombin receptors, protease-activated receptors (PARs) (Aoki T, et al. Thromb Haemost. 1998;79:1184-1190). As results using laser scattering system, when the platelets were stimulated in combination with TRAP (7 μM) and CXCL12 compared with TRAP alone, the increase of transmittance or the size ratio of large aggregates was not detected whereas the size ratios of small and medium aggregates were increased. The representative results are presented as Fig. 2A in the re-revised manuscript. In addition, when the platelets were stimulated in combination with ADP (0.5 μM) and CXCL12 compared with ADP alone, the increase of transmittance or the size ratio of large aggregates was not detected whereas the size ratios of small and medium aggregates were increased. The representative results are presented as Fig 2B in the re-revised manuscript. These findings suggest that the synergistic effects of thrombin or ADP with CXCL12 at subthreshold doses on platelet aggregation were hardly observed. Therefore, it is probable that the synergistic effect of CXCL12 on human platelet aggregation is specific to collagen. However, it has been reported that CXCL12 and TRAP as well as collagen synergistically stimulate platelet aggregation in mice (Karim ZA, et al. Biochim Biophys Acta 2016;1863:314-321). The discrepancy between the present and previous findings might be due to differences in species or experimental conditions.

To further define the receptor for collagen in the synergistic effect of collagen and CXCL12 on human platelet aggregation, we examined the platelet aggregation stimulated with a combination of CXCL12 and convulxin. Convulxin is a snake venom activator of GPVI but not integrin α2/β3 (Niedergang F, et al. Biochem Biophys Res Commun. 2000;273:246-250). We found that convulxin at a dose of 20 ng/ml had little effect on human platelet aggregation whereas 30 ng/ml of convulxin alone stimulated the aggregation. As results, the combination of convulxin (20 ng/ml) and CXCL12 (10 ng/ml) synergistically stimulated the platelet aggregation. Therefore, these findings suggest that GPVI among collagen receptors is involved in the synergistic effect with CXCL12 on human platelet aggregation. The representative results are presented as Fig. 3 in the re-revised manuscript.

As you pointed out, it is generally recognized that CXCL12 binds to both CXCR4 and CXCR7 receptors on human platelets. Therefore, in order to define the receptor for CXCL12 in the synergistic effect of collagen and CXCL12 on the platelet aggregation, we examined whether anti-CXCR4 antibody and/or anti-CXCR7 antibody could neutralize the putative synergistic effect of CXCL12 with collagen on the platelet aggregation or not. The anti-CXCR4 antibody markedly suppressed the platelet aggregation stimulated by a combination of CXCL12 and collagen. However, the synergistic effect of collagen and CXCL12 was hardly affected by anti-CXCR7 antibody. The representative results are presented as Fig. 4 in the re-revised manuscript. Regarding the exact mechanism underlying the synergistic effect of collagen and CXCL12 on human platelet aggregation, we showed that convulxin [33] mimicked the synergistic effect of collagen with CXCL12 on platelet aggregation, suggesting the involvement of GPVI as the receptor of collagen. Additionally, an antibody against CXCR4 but not CXCR7 suppressed the platelet aggregation induced by the simultaneous stimulation, suggesting the involvement of CXCR4 as the receptor for CXCL12. Based on our findings, it is most likely that the synergistic effect of CXCL12 and collagen on human platelet aggregation is mainly mediated through GPVI and CXCR4, the platelet membrane receptors for collagen and CXCL12, respectively.

We presented the data of additional experiments as Fig. 1, Fig. 2, Fig. 3 and Fig. 4, cited the above articles as Ref. 32, Ref. 37 and Ref. 33, respectively, and added the description in “Abstract”, “Materials and methods”, “Results” and “Discussion” in the re-revised manuscript. (page 2, line 38 to line 44, page 6, line 151 to page 7, line 158, page 8, line 188 to line 192, page 11, line 266 to line 278, page 12, line 290 to line 298, page 12, line 310 to page 13, line 321, page 23, line 571 to line 587)

**Specific comments**

1. **Table 1 and Figure 1 is redundant.**

Our responses:

To address your criticism that Table 1 and Figure 1 in the original manuscript was redundant, we deleted Table 1 appeared in the original manuscript from the re-revised manuscript.

**2. It is not clear why the authors used 10ng/ml of CXCL12 and 0.1µg/ml collagen. A dose-response curve would be of interest to validate the results. Is the synergistic effect of collagen and CXCL12 only found in the described and tested concentrations?**

Our responses:

As pointed out, we evaluated the dose-response curves of the synergistic effects of collagen and CXCL12 in human platelet aggregation. We examined the effect of the simultaneous stimulation of various doses (0.05 μg/ml, 0.1 μg/ml and 0.2 μg/ml) of collagen with a fixed dose (10 ng/ml) of CXCL12 on the platelet aggregation. As results, whereas collagen at a dose of up to 0.1 μg/ml with CXCL12 hardly increased the transmittance or size ratio of large aggregates, collagen at a dose of 0.2 μg/ml with CXCL12 significantly induced platelet aggregation. The representative results are presented as Fig. 1A in the re-revised manuscript. We further examined the effect of the simultaneous stimulation of various doses (1 ng/ml, 3 ng/ml and 10 ng/ml) of CXCL12 with a fixed dose (0.2 μg/ml) of collagen on the platelet aggregation. As results, whereas CXCL12 up to 3 ng/ml with collagen had little effect on the transmittance or size ratio of large aggregates, CXCL12 at a dose of 10 mg/ml with collagen significantly induced platelet aggregation. The representative results are presented as Fig. 1B in the re-revised manuscript. From the dose-response reactions of collagen and CXCL12, it is probable that the synergistic effect of collagen and CXCL12 in the combination of 0.2 μg/ml of collagen and 10 ng/ml of CXCL12 is prominent to induced the platelet aggregation, whereas the combination of these stimulators at lower doses is insufficient.

We presented the data of these additional experiments as Fig 1, and added the description in “Results” and “Discussion” in the re-revised manuscript. (page 10, line 240 to line 252, page 23, line 567 to line 571)

According to the revision, we deleted the data presented as Fig. 1 in the original manuscript.

2. **Fig 2B to Fig 2D should be combined for sake of clarity.**

Our responses:

As pointed out, we combined Fig. 2A, Fig.2B, Fig. 2C and Fig. 2D in the original manuscript into a panel as Fig. 5 in the re-revised manuscript.

**4. The discussion should be revised in regard a clear pathophysiological hypothesis where the described findings may play a role.**

Our responses:

We really appreciate your constructive suggestion. To address your comment, we revised the discussion in regard a clear pathophysiological hypothesis including a role of HSP27 in the re-revised manuscript as follows:

The progression of atherosclerotic pathology, therefore, might be also accelerated by the release of HSP27 from platelets, which could stimulate the vascular smooth muscle cell mitogenesis and the inflammatory responses of vascular endothelium cells in cooperation with PDGF-AB and sCD40L. (page 26, line 661 to line 664)

**-Reviewer 3**

**The manuscript is general sheds light on an important aspect of platelet biology, one in which CXCL12 plays a critical role in synergizing with collagen. We do have some issues that need to be addressed.**

**1. The “English” in the manuscript requires a lot of work, in terms of grammar and typos.**

Our responses:

According to your comment, the revised manuscript was thoroughly checked by a native English speaker.

**2. Abstract implies CXCL12 acts via CXCR only, whereas introduction via CXCR4 and CXCR7. This appears contradictory.**

Our responses:

According to your comment, we added “CXCR7” to the sentences in “Abstract” in the re-revised manuscript. (page 2, line 30)

**3. Elaborate on why the sCD40L assay the co-stimulation lasted for 15 minutes, unlike many of the other experiments.**

Our responses:

As pointed out, we adopted the co-stimulation lasted for 15 min in the sCD40L assay. Regarding the mechanism of sCD40L release from human platelets, CD40 ligand appears on the surface of activated platelets, and undergoes cleavage to release sCD40L (Davi G, *et al.* N Engl J Med. 2007;357:2482-2494, cited as Ref. 6 in the original manuscript). On the other hand, PDGF-AB is secreted on time from α-granule accompanied with the platelet aggregation. In addition, we have previously reported that the release of sCD40L from activated platelets requires more than 5 min and reaches to the plateau up to 30 min after the stimulation (Enomoto Y, *et al.* Atherosclerosis. 2010;209:415-421). Therefore, we determined the experimental condition as presented. To be more understandable, we cited the above article as Ref. 34, and added the description in “Results” in the re-revised manuscript as follows:

Regarding the mechanism of sCD40L release from human platelets, CD40 ligand appears on the surface of activated platelets, and undergoes cleavage to release sCD40L [6]. On the other hand, PDGF-AB is secreted on time from α-granule accompanied with the platelet aggregation. We have previously reported that the release of sCD40L from activated platelets requires more than 5 min and reaches to the plateau up to 30 min after the stimulation [34]. Therefore, we adopted the co-stimulation lasted for 15 min in the release of sCD40L, whereas the co-stimulation for 5 min in the secretion of PDGF-AB. (page 14, line 341 to line 349)

**4. SB203580 did not completely block the co-stimulation effects, which suggests a role for another pathway. What is it? Can you answer that experimentally?**

Our responses:

As pointed out, SB203580 did not completely block the effect of simultaneous stimulation with collagen and CXCL12, suggesting a role for another pathway. Unfortunately, we could not answer that experimentally at this time. To address your comment, we added the description in “Discussion” in the re-revised manuscript as follows:

The suppression by SB203580 of the effect of simultaneous stimulation with collagen and CXCL12 on the platelet activation was not complete, suggesting that pathway(s) in addition to p38 MAPK might be involved in the synergistic effect. (page 24, line 600 to line 603)

**5. All of the Western blot data should be quantified.**

Our responses:

We agreed to your criticism that all of Western blot data should be quantified. To address the comment, we quantified the Western blot data presented in Fig. 4, Fig. 5 and Fig. 9A in the original manuscript, and remade them including the bar graph of quantification in Fig. 7, Fig. 8 and Fig. 12A in the re-revised manuscript.

**6. Another relevant article not cited is by Karim et al, 2016 in BBA (PMID: 26628381). It should be cited.**

Our responses:

We really appreciate your suggestion. According to your suggestion, we cited the article (Karim ZA, *et al.* Biochim Biophys Acta. 2016;1863:314-321) as Ref. 37 and added the description in “Discussion” in the re-revised manuscript as follows:

However, it has been reported that CXCL12 and TRAP as well as collagen synergistically stimulate platelet aggregation in mice [37]. The discrepancy between the present and previous findings might be due to differences in species or experimental conditions. (page 23, line 574 to line 577)

According to the revision, Fig. 3, Fig. 4, Fig. 5, Fig. 6, Fig. 7, Fig 8A, Fig. 8B, Fig, 9A, Fig. 9B, Fig. 9C in the original manuscript were renamed to Fig. 6, Fig. 7, Fig. 8, Fig. 9, Fig. 10, Fig. 11A, Fig. 11B, Fig. 12A, Fig. 12B and Fig. 12C in the re-revised manuscript respectively, and Fig.2A, Fig.2B, Fig2C and Fig. 2D in the original manuscript were combined to Fig. 5 in the re-revised manuscript. Additionally, Ref. 32 and Ref. 33 in the original manuscript were renumbered as Ref. 35 and Ref. 36 in the re-revised manuscript. Ref. 34, Ref. 35, Ref. 36, Ref. 37, Ref. 38, Ref. 39, Ref. 40 and Ref. 41 in the original manuscript were renumbered as Ref. 38, Ref. 39, Ref. 40, Ref. 41, Ref. 42, Ref. 43, Ref. 44 and Ref. 45 in the re-revised manuscript.
